# Speech- and Language-Based Classification of Alzheimer’s Disease: A Systematic Review

**DOI:** 10.3390/bioengineering9010027

**Published:** 2022-01-11

**Authors:** Inês Vigo, Luis Coelho, Sara Reis

**Affiliations:** 1 Instituto Superior de Engenharia do Porto, 4249-015 Porto, Portugal; 1170969@isep.ipp.pt (I.V.); ssr@isep.ipp.pt (S.R.); 2 Centro de Inovação em Engenharia e Tecnologia Industrial, 4249-015 Porto, Portugal

**Keywords:** Alzheimer’s disease (AD), speech, classification, features, machine learning (ML), mild cognitive impairment (MCI)

## Abstract

Background: Alzheimer’s disease (AD) has paramount importance due to its rising prevalence, the impact on the patient and society, and the related healthcare costs. However, current diagnostic techniques are not designed for frequent mass screening, delaying therapeutic intervention and worsening prognoses. To be able to detect AD at an early stage, ideally at a pre-clinical stage, speech analysis emerges as a simple low-cost non-invasive procedure. Objectives: In this work it is our objective to do a systematic review about speech-based detection and classification of Alzheimer’s Disease with the purpose of identifying the most effective algorithms and best practices. Methods: A systematic literature search was performed from Jan 2015 up to May 2020 using ScienceDirect, PubMed and DBLP. Articles were screened by title, abstract and full text as needed. A manual complementary search among the references of the included papers was also performed. Inclusion criteria and search strategies were defined a priori. Results: We were able: to identify the main resources that can support the development of decision support systems for AD, to list speech features that are correlated with the linguistic and acoustic footprint of the disease, to recognize the data models that can provide robust results and to observe the performance indicators that were reported. Discussion: A computational system with the adequate elements combination, based on the identified best-practices, can point to a whole new diagnostic approach, leading to better insights about AD symptoms and its disease patterns, creating conditions to promote a longer life span as well as an improvement in patient quality of life. The clinically relevant results that were identified can be used to establish a reference system and help to define research guidelines for future developments.

## 1. Introduction

### 1.1. Context and Objectives

Alzheimer’s Disease (AD) is currently the most common cause of dementia from neurodegeneration all over the world, contributing to 60–70% of all cases. In 2006, the worldwide prevalence of AD was 26.6 million and, by 2050, the prevalence is predicted to reach 131 million, resulting in 1 in every 83 people in the world living with the disease [1,2]. Early and accurate diagnosis of AD has a major impact on its progress and follow-up, and although memory loss and behavioral changes are relevant indicators for its detection, these only become evident in more advanced stages of the disease, often leading to the late diagnosis of dementia [3,4]. Neuropsychological tests, an alternative to more expensive and often invasive approaches, can be powerful indicators of converting patients (from mild cognitive disease to AD), in particular when machine learning approaches are used [5,6]. In a systematic review, encompassing neuropsychological measures [7], categorical fluency tests for language, covering executive control ability and verbal ability, showed the highest performance when discriminating between healthy controls and Alzheimer’s, and measures of linguistic abilities achieved a high level of accuracy (ranging from 0.84 to 0.93). Hence, the loss of language skills and the ability to communicate, are common symptom among people with dementia, and can be used as relevant biomarkers.

Classification of AD based on language and speech is a relatively new field, and so far, there are no established and widely accepted methods when we considering a computational/algorithmic perspective. This review aims to recognize best and common practices, and bring together the most important aspects when developing such systems, covering acoustic levels but also linguistic levels as phonological, semantic, morpho-syntactic and pragmatic. For this, a set of scientific articles, obtained using a keyword search on scientific repositories, in the field of Alzheimer’s characterization using acoustic and linguistic voice patterns, has been reviewed. The main existing speech databases (or other resources or records) were listed and characterized as suitable for the intended purpose; the most commonly used discriminative features, which allow for the best results, alone or combined, are presented; the most recurrent and best performing machine learning (ML) models were also listed.

This review is highly focused on technical aspects of computational systems, and it is intended to help developers on the selection of resources and tools as well as defining the best architectures and approaches. The main question that we wanted to answer is, “When developing a computational system for classification of Alzheimer’s disease using speech and language, what resources are available and what approaches can lead to the best performance?”

### 1.2. Speech and Language Impairments in Alzheimer’s Disease

Alzheimer’s disease (AD) is one of the most devastating brain diseases in the world, especially in the more advanced age groups [8]. It is a progressive neurological disease that results in irreversible loss of neurons, particularly in the cortex and hippocampus, which leads to characteristic memory loss and behavioral changes in humans [9].

Although the nature of AD is unknown and is likely to be a multiple-cause disease, it has been observed that its onset is insidious and appears in adulthood, causing, in advanced stages, a cognitive and behavioral disability [10].

As the disease progresses, the quality of life of patients is deeply affected in different ways. As they lose cognitive abilities and functional skills, individuals with this dementia become unable to perform many of the activities that were usually part of their daily lives. Behavior and social skills may also deteriorate, precipitating interpersonal conflicts that lead to the individual with AD being socially isolated. This, in turn, has an impact on their emotional state [11]. In these syndromes, amnesic symptoms may not be the first evidence, but others, more prominent initial aspects, such as language problems, visual dysfunction, or difficulties with praxis [12].

Mild cognitive impairment (MCI) is known to be one of the first detectable indicators of cognitive decline. It is a heterogeneous syndrome that shows great clinical importance for the early detection of AD [13]. At this stage, the symptoms related with the ability to think begin to be noticed by the individual himself and by his closest members, but there are no functional changes in its daily life. Not all patients diagnosed with MCI develop AD, in fact, only 10 to 15% per year. There are two types of MCI, the amnesic and the non-amnesic. Patients with the first type are thought to have a greater tendency to develop AD. In cases where they do, MCI is considered the second phase of AD [14]. In general, the MCI captures the point in the spectrum of cognitive function between non-dementia aging and dementia with main characteristics for the amnesic type [15].

The general diagnosis of neurodegenerative diseases is usually compromised by the fact that the symptoms that trigger it represent an advanced stage of the disease, causing it to appear late. Therefore, the assessment of dementia should be based on four key issues: (1) whether there is a subjective disability detected by the individual himself or observed by a close individual; (2) whether there is objective evidence of cognitive disability in the tests performed; (3) whether there is a functional decline; (4) whether there are symptoms caused by something inherent in dementia (e.g., delirium, substances or other medical, neurological or psychiatric disorders). To answer these questions, a medical history is acquired, and appropriate physical examinations and laboratory studies are performed, as well as cognitive screenings, that also use neuroimaging techniques [15]. Within cognitive tests, it stands out the Mini-Mental State Exam (MMSE), the Clock-drawing test, and the Alzheimer’s Disease Assessment Scale [12,16,17]. The main exams using imaging techniques are Computed Axial Tomography (CT), Magnetic Resonance Imaging (MRI), Positron Emission Tomography (PET), and Single-Photon Emission Computed Tomography (SPECT) [15]. Although there is currently a wide range of diagnostic methods applied to AD, there is still a concern to find new methods that respond more urgently to dementia while being simple and cost effective.

Alzheimer’s disease is characterized by a progressive worsening of deficits in several cognitive fields, including language. Aphasia and dysarthria are common symptoms and language impairment in AD occurs mainly due to a decline in semantic and pragmatic levels of language processing [18]. From a physiological perspective, superior parietal, posterior temporal, and occipital cortical areas are interconnected by posterior corpus callosum. The superior longitudinal fasciculus surrounds the putamen, connecting all four cerebral lobes, areas that are known to be affected in MCI and AD and that have a central role in language processing [19,20]. Language difficulties are a major problem for most patients with dementia, especially as the disease progresses. The first signs that communication is being affected are the difficulties on finding words, especially when it comes to naming familiar people or objects. Words are replaced by wrong and meaningless words and pauses during speech are increased as well [21]. In the early stages of AD, language impairment involves problems of lexical recovery, loss of verbal fluency, and a breakdown in higher-order written and spoken language comprehension. In the moderate and severe phases of AD, the loss of verbal fluency is profound, with loss of understanding and prominent literal and semantic paraphrases. In the very severe phases of AD, speech is often restricted to echolalia and verbal stereotypes. In Table 1, it is possible to see the association of the mentioned speech impairments with the stage of the disease [18,22]. Communicative difficulties (speech and language) constitute one of the groups of symptoms that most accompany dementia and, therefore, should be recognized as a central study instrument. This recognition aims to provide earlier diagnosis, resulting in greater effectiveness in delaying the disease evolution.

Temporal and acoustics parameters, though less explored for AD, are also reported to change. Fundamental frequency, interruption of sound, voice periods, speech rate, among others, show distinct ranges in AD and healthy individuals [24,25,26]. Though they are out of the scope of this review, depression or mood changes, symptoms connected with AD, can also be classified using speech analysis.

## 2. Materials and Methods

**Figure 1:**
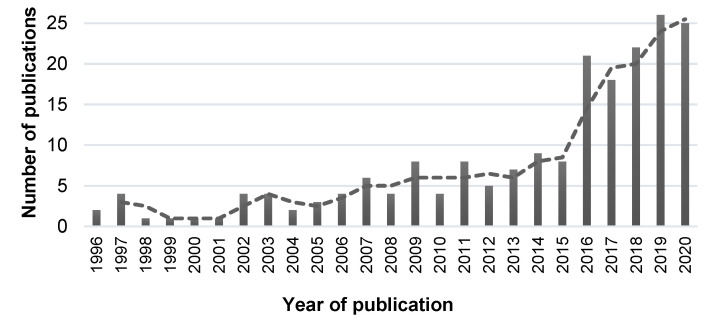
Number of publications (within the review’s scope) by year, in absolute value.

The methodology for this systematic review was inspired on the PRISMA (Preferred Reporting Items for Systematic reviews and Meta-Analyses) [27,28], registered with the number CRD42022296738 at the National Institute for Health Research (Prospero) database. ScienceDirect, PubMed, and DBLP scientific repositories, used as information sources, were searched through May 2020. Based on central keywords we have defined the as search query: (Alzheimer’s [Title] AND “Speech [Title] AND (“Detection [Title]” OR “Classification [Title]”)), that we have used similarly for each database. As eligibility criteria we have defined the following: (a) English language articles; (b) Published in peer-reviewed journal; (c) Related with machine learning or statistical methods; (d) Processing pipeline details were clearly defined. Using the first repository, as a preparatory step, a statistical analysis of the number of publications per year was made, from 1996 to May 2020. After a coarse removal of out-of-scope articles and duplicates, it was possible to count the number of publications per year, as presented in Figure 1. This allowed to observe a significant increase in the research interest in this topic since 2015, therefore, it was decided to restrict the analysis to the period from 2015 to 2020. In ScienceDirect, a filter was applied so that only research articles were displayed, and in DBLP two filters were applied simultaneously, so that it was possible to restrict the articles to those that were classified as academic journals and whose content was related to “machine learning”.

We have not assessed the risk of bias for these studies due to its great heterogeneity and differences in background scientific fields (some studies were clinical oriented, such as non-randomized studies or randomized controlled trials, while others were developed as exploratory machine learning exercises, with no pretension to immediate application in clinical decision). But we consider that, since many studies are based on stochastic approaches, bias risk should be better addressed in these articles, especially when creating speech databases, where gender, age, disease severity, comorbidities, among others, should be carefully balanced.

**Figure 2:**
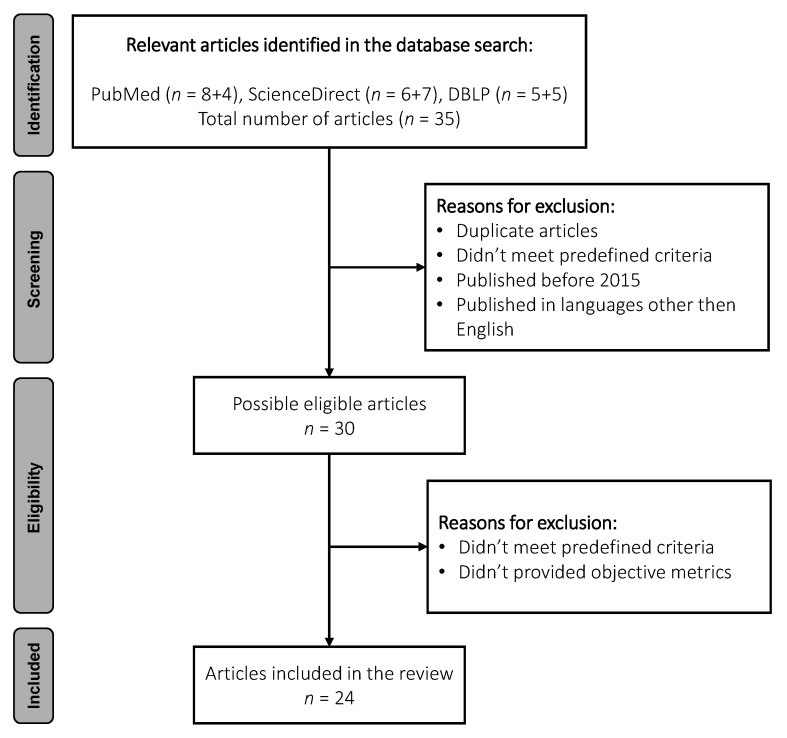
Flow chart of the different phases of the review.

After applying the filters, the articles of interest were selected manually. This process involved careful reading of the article’s abstract, where only those that approached the detection of AD or MCI based on speech and language, were selected. In a deeper analysis of the obtained articles, 14 duplicates were detected. In addition to the duplicates found, 2 more articles from the IEEE platform were added, by reference following in the first selected bibliography. Thus, the database created has 24 articles from the platforms mentioned. In Figure 2 it is possible to observe the process to reach this total number of articles. Finally, our search strategy, was focused on identifying the main components of machine learning and statistical-based approaches: data sources, data models, parameter optimization strategies; and on the outcomes provided by such systems: evaluation strategies and performance indicators.

## 3. Results

In this section we will present the outcomes of our literature review. We start by presenting the systems’ overall architecture and then, on each subsection, we will focus on the composing elements.

### 3.1. Machine Learning Pipeline

The use of speech analysis is potentially a useful, non-invasive, and simple method for early diagnosis of AD. The automation of this process allows a fast, accurate, and economical follow-up over time. Initially, speech-based tests for AD detection were performed by linguists. These tests were designed to extract linguistic characteristics from speech or writing samples. However, more current studies seek to optimize this task by automating the process of speech recognition through audio recordings [29]. Thus, and in sequence, the process can be described in 4 crucial steps:

1. Data Preparation: In this step the extraction, optimization and normalization of features occurs. This consists in the selection of the most significant features (by removal of the non-dominant features) and in the transformation of ranges to similar limits, which will reduce training time and the complexity of the classification models. Metadata are “the data of the data”, more specifically, structured, and organized information on a given object (in this case voice recordings) that allow certain characteristics of it to be known. This metadata together with the results of the pre-processing of the recordings makes the final database. Incorrect or poor-quality data (e.g., outliers, wrong labels, noise, …), if not properly cared for, will lead to under optimized models and to unsatisfactory results. If data is not enough, for example when deep learning algorithms are used, then data augmentation techniques can be useful.
2. Training and Validation: The supporting database is divided into subsets, usually 70–90% for training and 30–10% for testing. The subsets can be randomly generated several times and the results can be averaged for additional confidence in the results, a procedure that is designated by cross-validation. The data model is trained, i.e., the involved parameters are adjusted, by one or many optimizers, and the performance is calculated using the test subset. This step allows categorizing and organizing the data to promote better analysis [30]. When data is not enough, then transfer learning approaches can be used.
3. Optimization: After model evaluation, it is possible to conclude on the parameters that need to be improved, as well as to proceed in a more effective way to the selection of the most interesting and relevant features, so that a new extraction and consequently a new process (iteration) of Training and Validation can be performed.
4. Run-Time: Having concluded the previous points, the system is ready to be deployed and to classify new unseen inputs. More specifically, from the recording of a patient’s voice, to classify it as possible healthy or possible Alzheimer’s patient.

**Figure 3:**
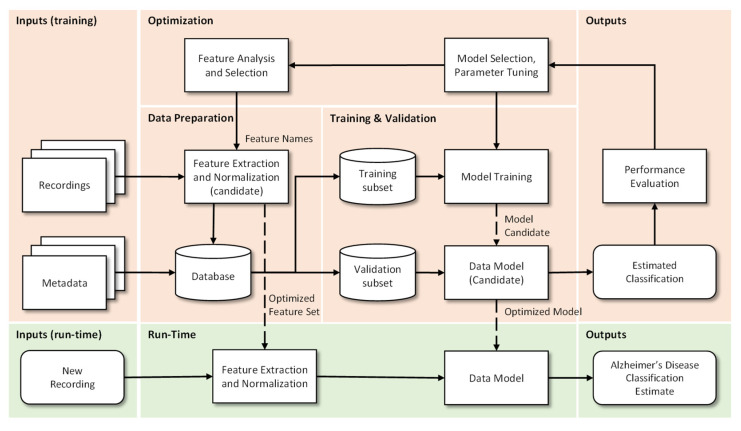
Flowchart of a general machine learning pipeline to process acoustic/prosodic correlates of disease. Adapted from Braga et al. [31].

In Figure 3 we can observe the described methodology in detail.

### 3.2. Speech and Language Resources

As mentioned above, to be able to create a mechanism for detecting AD, a speech database is required. Building a speech database implies careful planning. Important steps that should be followed and prepared in an initial design stage are: recording conditions, acquisition and storage hardware, data collection protocol, informant selection, speech task, data organization and labelling. As sensitive data can be collected, ethical and safety aspects should also be of concern. The quality of the database is crucial since it supports the analysis and the conclusions that can be drawn.

With the increasing interest on the area, the number of speech and language resources has also increased (although many languages are not yet covered). Table 2 presents the main databases that are referred in the scientific literature, accompanied by a summary of their characteristics. These resources are crucial for supporting the development of new systems, in particular when deep learning approaches are used. The use of similar databases in different studies, by different researchers, also provides a common ground for evaluation and performance comparison.

The BEA (whose acronym comes from BEszélt nyelvi Adatbázis) is a growing database containing various types of spontaneous speech, reading aloud, and conversation in Hungarian. To date, it consists of records of 280 healthy and cognitively declining subjects between the ages of 20 and 90 [56].

Cinderella contains recordings of 60 subjects spontaneously telling the story of Cinderella. These 60 subjects, Portuguese native speakers, are equally divided into the groups healthy, with MCI, and with AD. The records that make up the database were made by researchers Toledo et al. [45] for the study in question; the character of the database in terms of availability is undefined.

TalkBank is a project whose main objective is to encourage the study in the field of human communication. Currently, it makes available repositories of several research areas covering more than 34 languages, all of them open-source upon request. DementiaBank is one of the repositories that this project has, which as its name indicates, focuses on the communication of people with dementia. Within this repository, there are several Corpus with different languages, tasks, and dementias under analysis. In Table 2 and Table 3, there are two examples of the corpus that can be found in DementiaBank, Lu Corpus, and Pitt Corpus.

Dem@care is a European project focused on improving the quality of life of people with dementia. This project has multilingual databases and files of different types, such as audio and video. These databases are available upon request, and there is also a quick contact section on the website available at the footer. Although none of the studies had made use of this database, it is highly referenced in the literature covered.

The Gipuzkoa-Alzheimer Project (GAP) is a longitudinal Spanish study, running since 2011 where volunteers are observed every 3 years to analyze the evolution of the disease. The database that this study gathers can be accessed upon request [57].

The Wisconsin Registry for Alzheimer’s Prevention (WRAP) has been conducting a longitudinal study to assess parameters that allow early detection of cognitive decline at older ages. To date, 1561 people have participated in this study, who have been subjected to various types of analysis methods and continuously over several years. The WRAP protocol resources and databases of related studies can be accessed by qualified researchers by completing an online form and a data use agreement, which can be found on the Global Alzheimer’s Association Interactive Network website [58].

### 3.3. Language and Speech Features

As mentioned in Table 1, the most evident problems early on in AD, as far as speech is concerned, are related to difficulties in general semantics, that is, in finding words to name objects. In this sense, temporal cycles during spontaneous speech production (speech fluency) are affected and, therefore, can be detectable in the patient’s hesitation and pronunciation [59]. Other speech characteristics affected in AD patients seem to be those related to articulation (speed in language processing), prosody in terms of temporal and acoustic measurements, and eventually, in later phases, phonological fluency [60].

Considering the linearity of the features, they can be classified as linear or non-linear, the linear ones being more conventionally used. Linear features can be subdivided into several groups, but these are always very interconnected. Thus, we chose to divide into two groups, linguistics, and acoustics, and present them in Table 3 and Table 4. For each reviewed article we have collected the name of the features that were used.

The reviewed literature does not present an immediate pattern regarding the extraction and use of features, and it is possible to find simple sets based on traditional metrics, but also other approaches using advanced parameters and methods, using one or several feature sets. All studies report good accuracies and promising results.

Using linguistic features, Rentoumi et al. [40] developed studies for computational linguistic analysis in Alzheimer’s patients, resulting in maximum accuracies of 88%.

To identify changes in the macro-linguistic aspects of speech in subjects with cognitive decline, Toledo et al. [45] conducted a study, in Portuguese, where the history of Cinderella was used as the main task of analysis. Using, in the same way, linguistic features, it was possible to distinguish the various degrees of dementia.

The task of picture description is one of the most used for the analysis of spontaneous speech. A study carried out by Hernández-Domínguez et al. [61] uses this same task, proposing a new methodology that allows patients to be described, later allowing them to be classified as Alzheimer’s patient or not. This classification reached accuracies of 94% using linguistic features.

With the main objective of detecting MCI, Fraser et al. [51] developed two studies. The first, bilingual, which allowed the creation of a detection system applicable to two languages, English and Swedish, also allowing the evaluation of the impact of the language on the accuracy of this detection. The second has taken a cascade approach to combine data from multiple language tasks to distinguish patients with CCL and healthy patients, achieving 83% accuracy [51]. In both studies, the extracted features were linguistic.

Martínez-Sánchez et al. [49] presented a study to validate a prototype that automatically performs speech analysis in older people with AD. The device created, and based on acoustic features, provides numerical parameters that can be interpreted to identify specific changes in speech fluency, acoustics, and prosody, and was able to correctly classify 92.4% of the subjects under study. Also using acoustic features [13,52,62,63], achieved accuracies of 97%, 83%, 71.4%, and 62%, respectively.

Khodabakhsh et al. [54,55] conducted three studies in the area of focus. In the first two studies, acoustic features were used to detect AD, where accuracies of 94% were reported for both proposed approaches. The third study encompassed a more extensive set of features where acoustic and linguistic features were combined, resulting in 84% accuracy, for a distinct dataset [53].

Qiao et al. [44] created an automatic speech recognition software specialized in cognitive impairment, allowing the characterization of language impairment in people with AD and MCI. For this, they used acoustic features.

Alexandra König et al. [36] proposed to use several short cognitive vocal tasks to distinguish between healthy controls, mild cognitive impairment and AD patients, with the best distinction being between healthy subjects and Alzheimer’s patients, with an accuracy of 87%. The same authors also proposed a mobile application to record spontaneous speech in an uncontrolled environment that proved to be an useful tool in providing additional indicators for early assessment and detection of AD and MCI [37]. By combining acoustic features in a semantic verbal fluency analysis, aimed at automating this process, the authors were capable of successfully distinguishing patients in a healthy group from patients with AD and MCI [38].

Acoustic and linguistic features were also used by Gosztolya et al. [41]. The authors have developed independent systems for each set of features, with an accuracy 82%, for both cases. The combination of both feature sets allowed to rise the scores to 86%, showing the importance of acoustic and linguistic information.

With the combination of acoustic features and linguistic features, two studies were conducted, one by Gosztolya et al. [41] and the other by Beltrami et al. [42], which obtained accuracies of 86% and 77%, respectively.

Chien et al. [43] have also developed a system for the analysis of AD through speech. However, contrary to what happens in most studies, the features instead of being selected by statistical methods were selected through an acoustic feature sequence generator created and trained as part of the proposed system.

Other unconventional features sets have also been used with interesting results. For example in [47,48] non-linear features are used, namely the fractal dimension and entropy of permutation that allowed reaching accuracies of 90.9%.

### 3.4. Classification Models

The process of classification lies in identifying to which, of a given set of categories, a new observation belongs to, based on another set of training categories whose observations have already been assigned a category [64]. Thus, after the extraction and selection of the most significant features, it is necessary to proceed to their classification so that it is also possible to classify the groups of data under study.

When data distribution or patterns are known, then a compatible model (linear, polynomial, exponential or other) will lead to optimal results. However, machine learning has gained special relevance due to its ability to provide good estimates even when facing unstructured high dimensionality data. In this context, deep neural networks (DNN) can excel. These are flexible models where elements, inspired on the human brain anatomophysiology, are combined in large structures, with several sequential layers, to provide the output. The number of elements per layer, the number of layers, and the behavior of each layer (fully connected, convolutional, recurrent, …) are some of the parameters that can be adjusted to fit the network to the data/problem. Despite the widespread use of these techniques, the high amount of training data that is required for training the huge number of parameters and the “black-box” model that is obtained in the end, are some of the often-mentioned caveats.

In Table 5, some of the most commonly used models are summarized and defined in general terms.

**Figure 4:**
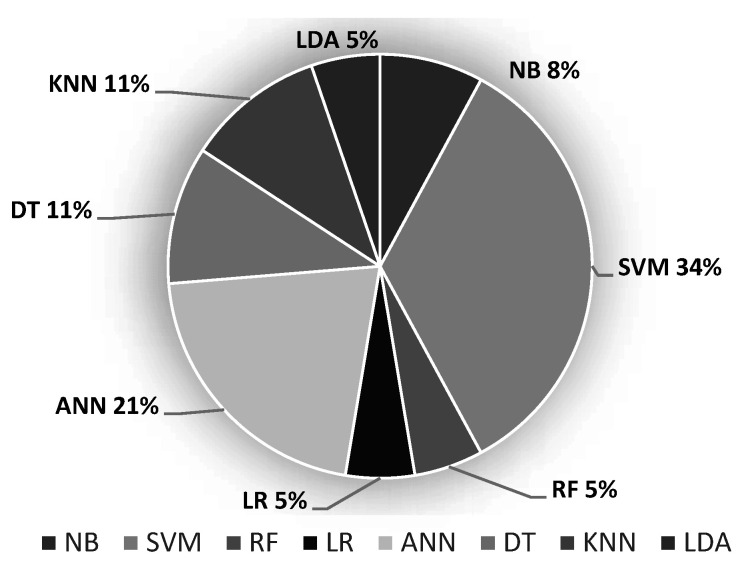
Prevalence of classification models.

Based on Table 5, it is possible to determine the frequency of use of each model, as can be seen in Figure 4. We can observe that the most popular classification models are based on Vector Support Machine (SVM), with 34%, followed by the several variations of Artificial Neural Networks (ANN), with 21%. The ability to deal with non-linear data distributions and possibility of finding non-obvious patterns in data may be the main motivations for their use.

### 3.5. Testing and Performance Indicators

To conclude on the efficiency and viability of the classification model adopted, it is necessary to evaluate it. To be able to compare the performance of a given system against others reported systems it is important to choose a common metric with a well/defined testing method/setup otherwise it will be impossible to understand how good a system stands against its competitors. In this sense, Table 6 presents the evaluation models applied in the literature search.

**Figure 5:**
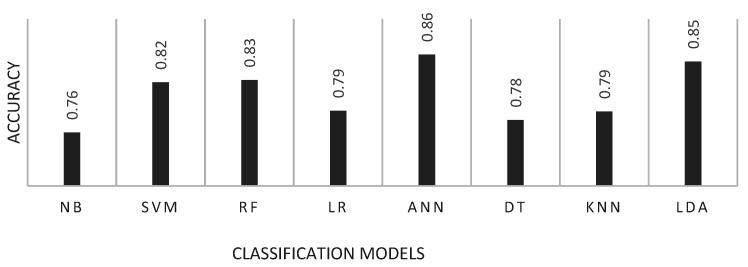
Mean accuracy by classification model.

Accuracy, among other metrics, is an indicator of quality that allows one to objectively evaluate the performance of systems, either alone or by comparison. Other common parameters of interest are the Area Under Curve (AUC) and the F1 score. However, accuracy is one of the preferred metrics and its value is provided by most authors. Figure 5 shows, for each classification model, the average accuracy values that was reported in the revised articles.

## 4. Discussion

Speech analysis, in general, represents an important source of information encompassing the phonetic, phonological, lexical-semantic, morphosyntactic, and pragmatic levels of language organization [72]. The first signs of cognitive decline are quite present in the discourse of neurodegenerative patients so that diagnosis via speech analysis of these patients is a viable and effective method, which may even lead to an earlier and more accurate diagnosis.

The reviewed articles focused on various aspects of identification or classification of cognitive loss. In terms of the evolution of the disease, it is possible to apply the techniques based on speech assessment in several stages: (a) in the area of early diagnosis; (b) in the classification/distinction between pathological cases and healthy individuals; (c) in the quantification symptoms intensity; (d) in the follow-up of the disease, characterizing the effectiveness of therapeutic approaches.

Further research is required to improve the systems performance and reliability.

### 4.1. Base Model for System Development

Despite the distinct objectives of the articles included in this revision it was possible to identify common modules, similar resources and shared methodologies. A base system, with a robust development base and with flexibility for exploration, should follow:

- DATABASE. The DementiaBank database, provided by the TalkBank platform, would be used due to its versatility in terms of population, types of tasks, and languages; This is robust resource, widely known and used, that can be useful when comparing systems using a common linguistic base.
- FEATURES. A combination of linguistic and acoustic features seems to provide the best results, namely the duration and the total number of silences, voice segments, and hesitations, as well as the fundamental frequency, jitter, and shimmer, as they are of the characteristics where a greater difference between healthy individuals and individuals with AD.
- TASK. Given the previously mentioned features, spontaneous speech would be used as the main task for assessment, using questions that would generate a fluent and spontaneous conversation.
- CLASSIFICATION MODELS. As classification models, Artificial Neural Networks should constitute the base model for decision due to their flexibility to data patterns and because the provide a high dimension parameter space that can be explored and tuned. Systems based on these models have the highest reported accuracies.
- EVALUATION MODELS. As it is the most recurrent, cross-validation should be applied to evaluate the classification models. Accuracy and F-score should be the comparison metrics.

The integration of the modules and the tuning of the final system are also a matter of concern. Closed-loop systems, that can automate parameter search are of great interest when designing a machine learning tool. A better performance system ensures that the subject’s final rating is more reliable and safer. That said, although these systems are a possible way of detecting and classifying AD, it is important to note that their purpose was to help on an assisted diagnosis process. None of the reported system was evaluated as a clinical tool and the official diagnosis should be made by a specialist doctor. However, they demonstrate an added value in the sense that they assume the role of a time-saver, leading to people being diagnosed earlier and more quickly, also raising awareness of potential age groups who may go to visit a neurologist.

### 4.2. Future Work

With the evolution of technology also the methods of diagnosis and analysis are evolving. Thus, more, and better ways of detecting diseases or even new diagnostic processes are appearing. The detection and classification of Alzheimer’s disease, which was usually performed via neurological tests and neuroimaging, is now possible through less invasive and equally efficient methods. The existing models for the detection of AD through speech have been increasing in quantity and in quality, though improvements are still needed. At present, the biggest barriers in the methods created for the automatic detection of AD lie in the fact that: (a) most systems are language dependent; (b) the number of samples used per study is very small, so the number of experiments on which the system is based is little for it to achieve optimal performance; (c) System components are not always integrated and may require human intervention; (d) feature sets are not yet fully established although temporal aspects (total duration, speech rate, articulation rate, among others) pitch, voice periods and interruptions, when combined with language or linguistic features can lead to very good results. Additional research is needed to find the optimal combination of parameters and what tasks should the (potential) patient be invited to perform. Thus, it is envisioned as future work the implementation of multilingual or language independent systems, supported by extensive and diverse databases (that still must be gathered, with balanced number of M/F, ages, disease severity), as well as the automation of the features selection and extraction. Better decision models, task oriented, are also required.

## Figures and Tables

**Table 1:** Language changes in AD (adapted from Ferris and Farlow [18] and Greta et al. [23]).

Function	Early Stages	Moderate to Severe Stages
Spontaneous Speech	Fluent, grammatical	Non-fluent, echolalic
Paraphrastic errors	Semantics	Semantic and phonetic
Repetition	Intact	Very affected
Naming objects	Slightly affected	Very affected
Understanding the words	Intact	Very affected
Syntactical understanding	Intact	Very affected
Reading	Intact	Very affected
Writing	± Intact	Very affected
Semantic knowledge of words and objects	Difficulties with less used words and objects.	Very affected

**Table 2:** List of databases, with related specifications, with Alzheimer’s patients’ speech recordings. (Table contents are sorted by language, first column, and database name, second column).

Language	Database Name	Task	Population			Availability	Refs.
			HC M/F	MCI M/F	AD M/F		
English	DementiaBank (TalkBank)	DF	99	-	169	Upon request	[32]
English	Pitt Corpus	PD	75/142	27/16	87/170	Upon request	[33]
English	WRAP	PD	59/141	28/36	-	Upon request	[34]
English	-	PD	112	-	98	Undefined	[35]
French	-	Mixed	6/9	11/12	13/13	Undefined	[36]
French	-	VF, PD, SS Counting	-	19/25	12/15	Undefined	[37]
French	-	VF, Semantics	5/19	23/24	8/16	Undefined	[38]
French	-	Reading	16	16	16	Undefined	[39]
Greek	-	PD	16/14	-	13/17	Undefined	[40]
Hungarian	BEA	SS	13/23	16/32	-	Upon request	[13] [41]
			25	25	25		
Italian	-	Mixture	48	48	-	Undefined	[42]
Mandarin	Lu Corpus	PD/SS	4/6	-	6/4	Upon request	[43]
Mandarin	-	PD/SS	24	20	20	Undefined	[44]
Portuguese	Cinderella	SS	20	20	20	Undefined	[45]
Spanish	AZTITXIKI (AZTIAHO)	SS	5	-	5	Undefined	[46]
Spanish	AZTIAHORE (AZTIAHO)	SS	11/9	-	8/12	Undefined	[47,48]
Spanish	PGA-OREKA	VF	26/36	17/21	-	Upon request	[47]
	Mini-PGA	PD	4/8	-	1/5		
Spanish	-	Reading	30/68	-	14/33	Undefined	[49]
Swedish	Gothenburg	PD	13/23	15/16	-	Undefined	[50]
Swedish	-	Mixed	12/14	8/21	-	Upon request	[51]
Swedish	-	Reading	11/19	12/13	-	Undefined	[52]
Turkish	-	SS/Interview	31/20	-	18/10	Undefined	[53]
Turkish	-	SS/Interview	12/15		17/10	Undefined	[54]
Turkish	-	SS	12/15	-	17/10	Undefined	[55]

Legend: M: Males; F: Females; HC: Healthy Controls; MCI: Mild Cognitive Impairment; AD: Alzheimer’s Disease; SS: Spontaneous Speech; VF: Verbal Fluency; PD: Picture Description; PGA: Gipuzkoa Alzheimer Project; WRAP: Wisconsin Registry for Alzheimer’s Prevention.

**Table 3:** Linguistic features that have been used for AD detection. The features are organized by type. For each feature name, the number of occurrences/usages is provided inside parenthesis.

Feature Type	Feature Name
Occurrence frequency	Words (3); Verbs (2); Nouns, Predicates (1); Coordinate and Subordinate Phrases (2); Reduced phrases (2); Incomplete Phrases/Ideas (3); Filling words (1); Unique words (2); Revisions/Repetitions (1); Word Replacement (2)
Time/Duration	Total speech (3); Speech Rate (3); Speech time (2); Average of syllables (2); Pauses (4); Maximum pause (2).
Parts of speech ratio	Nouns/Verbs (2); Pronouns/Substantives (1); Determinants/Substantives (2); Type/Token (2); Silence/Speaking (4); Hesitation/Speaking (3).
Semantic density	The density of the idea (1); Efficiency of the idea (1); Density of information (2); Density of the sentences (1).
POS (Parts-of-Speech)	Text tags (4).
Complexity	The entropy of words (1); Honore’s Statistics (1).
Lexical Variation	Variation: nominal (2), adjective (1), modifier (1), adverb (1), verbal (1), word (1); Brunet’s Index (1).

**Table 4:** Acoustic features that have been used for AD detection. The features are organized by type. For each feature name, the number of occurrences/usages is provided inside parenthesis.

Feature Type	Feature Name
Hesitations	Filled Pauses (2); Silent Pauses (4); Long Pauses (3); Short Pauses (3); Voice Breaks (5).
Voice Segments	Period (4); Average duration (4); Accentuation (2).
Frequency	Fundamental frequency (8); Short term energy (7); Spectral centroid (1); Autocorrelation (2); Variation of voice frequencies (2).
Regularity	Jitter (11); Shimmer (11); Intensity (6); Square Energy Operator (1); Teager-Kaiser Energy Operator (1); Root Mean Square Amplitude (2).
Noise	Harmonic-Noise ratio (3); Noise-Harmonic ratio (2).
Phonetics	Articulation dynamics (1); the rate of articulation (1); Pause rate (5).
Intensity	From the voice segments (1); From the pause segments (1);
Timbre	Formant’s Structure (6); Formant’s Frequency (8).

**Table 5:** Most significantly used classification models.

Model		Characterization	References
NB		Consists of a network, composed of a main node with other associated descending nodes that follow Bayes’ theorem [65].	[13,35,40,53]
SVM		Consists of building the hyperplane with maximum margin capable of optimally separating two classes of a data set [65].	[13,37,38,39,40,41,50,51,52,53,54,55,61,66]
RF		Relies on the creation of a large number of uncorrelated decision trees based on the average random selection of predictor variables [67].	[13,61]
DT		Consists of building a decision tree where each node in the tree specifies a test on an attribute, each branch descending from that node corresponds to one of the possible values for that attribute, and each leaf represents class labels associated with the instance. The instances of the training set are classified following the path from the root to a leaf, according to the result of the tests along the path [68].	[39,53,54,55]
KNN		Based on the memory principle in the sense that it stores all cases and classifies new cases based on similar measures [65].	[42,46,48]
LR		A model capable of finding an equation that predicts an outcome for a binary variable from one or more response variables [69].	[42,51]
LDA		It is a discriminatory approach based on the differences between samples of certain groups. Unsupervised learning technique where the objective is to maximize the relationship between the variance between groups and the variance within the same group [70].	[54,55]
ANN	DNN	Naturally inspired models. Supervised learning approach based on a theory of association (pattern recognition) between cognitive elements [71]. There are many possibilities with different elements, structures, layers, etc. The larger the number of parameters then the larger the dataset must be.	[42,43,46,47,48,52,53]
	CNN		
	RNN		
	MLP		

NB: Naive Bayes; RF: Random Forest; LDA: Linear Discriminant Analysis; SVM: Support Vector Machine; DT: Decision Trees; ANN: Artificial Neural Networks; RNN: Recurrent Neural Network; CNN: Convolutional Neural Networks; MLP: Multilayer Perceptron; KNN: k-Nearest Neighbors; DNN: Deep Neural Networks; LR: Logistic Regression.

**Table 6:** Evaluation models for classification models.

Model	Method	Reference
Cross Validation	k-Fold	[40,41,43,46,47,48,52,61]
	Leave-pair-out	[51,66]
	Leave-one-out	[13,38,50,53,54,]
Split Evaluation	90–10%	[52]
	80–20%	[42]
Random Sub-Sampling	-	[37]

## Data Availability

Not applicable.

## References

[1] Brookmeyer R., Johnson E., Ziegler-Graham K., Arrighi H.M. (2007). Forecasting the Global Burden of Alzheimer’s Disease. Alzheimer’s Dement..

[2] Prince M., Bryce R., Albanese E., Wimo A., Ribeiro W., Ferri C.P. (2013). The Global Prevalence of Dementia: A Systematic Review and Metaanalysis. Alzheimer’s Dement..

[3] Khachaturian Z.S. (1985). Diagnosis of Alzheimer’s Disease. Arch. Neurol..

[4] Weller J., Budson A. (2018). Current Understanding of Alzheimer’s Disease Diagnosis and Treatment. F1000Res.

[5] Pereira T., Ferreira F.L., Cardoso S., Silva D., de Mendonça A., Guerreiro M., Madeira S.C., for the Alzheimer’s Disease Neuroimaging Initiative (2018). Neuropsychological Predictors of Conversion from Mild Cognitive Impairment to Alzheimer’s Disease: A Feature Selection Ensemble Combining Stability and Predictability. BMC Med. Inform. Decis. Mak..

[6] Belleville S., Fouquet C., Hudon C., Zomahoun H.T.V., Croteau J., Consortium for the Early Identification of Alzheimer’s disease-Quebec (2017). Neuropsychological Measures That Predict Progression from Mild Cognitive Impairment to Alzheimer’s Type Dementia in Older Adults: A Systematic Review and Meta-Analysis. Neuropsychol. Rev..

[7] Battista P., Salvatore C., Berlingeri M., Cerasa A., Castiglioni I. (2020). Artificial Intelligence and Neuropsychological Measures: The Case of Alzheimer’s Disease. Neurosci. Biobehav. Rev..

[8] Soldan A., Gazes Y., Stern Y. (2017). Alzheimer’s Disease☆. Reference Module in Neuroscience and Biobehavioral Psychology.

[9] Nussbaum R.L., Ellis C.E. (2003). Alzheimer’s Disease and Parkinson’s Disease. N. Engl. J. Med..

[10] Pulido M.L.B., Hernández J.B.A., Ballester M.Á.F., González C.M.T., Mekyska J., Smékal Z. (2020). Alzheimer’s Disease and Automatic Speech Analysis: A Review. Expert Syst. Appl..

[11] Logsdon R.G., Gibbons L.E., McCurry S.M., Teri L. (1999). Quality of Life in Alzheimer’s Disease: Patient and Caregiver Reports. J. Ment. Health Aging.

[12] McKhann G.M., Knopman D.S., Chertkow H., Hyman B.T., Jack C.R., Kawas C.H., Klunk W.E., Koroshetz W.J., Manly J.J., Mayeux R. (2011). The Diagnosis of Dementia Due to Alzheimer’s Disease: Recommendations from the National Institute on Aging-Alzheimer’s Association Workgroups on Diagnostic Guidelines for Alzheimer’s Disease. Alzheimer’s Dement..

[13] Toth L., Hoffmann I., Gosztolya G., Vincze V., Szatloczki G., Banreti Z., Pakaski M., Kalman J. (2018). A Speech Recognition-Based Solution for the Automatic Detection of Mild Cognitive Impairment from Spontaneous Speech. Curr. Alzheimer Res..

[14] Alberdi A., Aztiria A., Basarab A. (2016). On the Early Diagnosis of Alzheimer’s Disease from Multimodal Signals: A Survey. Artif. Intell. Med..

[15] Wang L.Y., LaBardi B.A., Raskind M.A., Peskind E.R., Hantke N., Etkin A., O’Hara R. (2020). Chapter 14—Alzheimer’s Disease and Other Neurocognitive Disorders. Handbook of Mental Health and Aging.

[16] Cacho J., Benito-León J., García-García R., Fernández-Calvo B., Vicente-Villardón J.L., Mitchell A.J. (2010). Does the Combination of the MMSE and Clock Drawing Test (Mini-Clock) Improve the Detection of Mild Alzheimer’s Disease and Mild Cognitive Impairment?. J. Alzheimers Dis..

[17] Hancock P., Larner A.J. (2011). Test Your Memory Test: Diagnostic Utility in a Memory Clinic Population. Int. J. Geriatr. Psychiatry.

[18] Ferris S.H., Farlow M. (2013). Language Impairment in Alzheimer’s Disease and Benefits of Acetylcholinesterase Inhibitors. Clin. Interv. Aging.

[19] Zhang Y., Schuff N., Camacho M., Chao L.L., Fletcher T.P., Yaffe K., Woolley S.C., Madison C., Rosen H.J., Miller B.L. (2013). MRI Markers for Mild Cognitive Impairment: Comparisons between White Matter Integrity and Gray Matter Volume Measurements. PLoS ONE.

[20] Axer H., Klingner C.M., Prescher A. (2013). Fiber Anatomy of Dorsal and Ventral Language Streams. Brain Lang..

[21] Banovic S., Zunic L.J., Sinanovic O. (2018). Communication Difficulties as a Result of Dementia. Mater. Sociomed.

[22] Soria Lopez J.A., González H.M., Léger G.C. (2019). Alzheimer’s Disease. Handb Clin. Neurol..

[23] Szatloczki G., Hoffmann I., Vincze V., Kalman J., Pakaski M. (2015). Speaking in Alzheimer’s Disease, Is That an Early Sign? Importance of Changes in Language Abilities in Alzheimer’s Disease. Front. Aging Neurosci..

[24] Meilán J.J.G., Martínez-Sánchez F., Carro J., López D.E., Millian-Morell L., Arana J.M. (2014). Speech in Alzheimer’s Disease: Can Temporal and Acoustic Parameters Discriminate Dementia?. Dement. Geriatr. Cogn. Disord..

[25] Campbell E.L., Fernández L.D., Raboso J.J., García-Mateo C. (2021). Alzheimer’s Dementia Detection from Audio and Language Modalities in Spontaneous Speech. IberSPEECH.

[26] Mahajan P., Baths V. (2021). Acoustic and Language Based Deep Learning Approaches for Alzheimer’s Dementia Detection From Spontaneous Speech. Front. Aging Neurosci..

[27] Tricco A.C., Lillie E., Zarin W., O’Brien K.K., Colquhoun H., Levac D., Moher D., Peters M.D.J., Horsley T., Weeks L. (2018). PRISMA Extension for Scoping Reviews (PRISMA-ScR): Checklist and Explanation. Ann. Intern. Med..

[28] Page M.J., McKenzie J.E., Bossuyt P.M., Boutron I., Hoffmann T.C., Mulrow C.D., Shamseer L., Tetzlaff J.M., Akl E.A., Brennan S.E. (2021). The PRISMA 2020 Statement: An Updated Guideline for Reporting Systematic Reviews. BMJ.

[29] Walker L., Schaffer J.D. (2020). The Art and Science of Machine Intelligence.

[30] Allen M., Cervo D. (2015). Multi-Domain Master Data Management.

[31] Braga D., Madureira A.M., Coelho L., Abraham A., Abraham A., Muhuri P.K., Muda A.K., Gandhi N. (2018). Neurodegenerative Diseases Detection Through Voice Analysis. Proceedings of the Hybrid Intelligent Systems.

[32] Boller F., Becker J. (2005). Dementiabank Database Guide.

[33] Becker J.T., Boller F., Lopez O.L., Saxton J., McGonigle K.L. (1994). The Natural History of Alzheimer’s Disease. Description of Study Cohort and Accuracy of Diagnosis. Arch. Neurol..

[34] Mueller K.D., Koscik R.L., Hermann B.P., Johnson S.C., Turkstra L.S. (2018). Declines in Connected Language Are Associated with Very Early Mild Cognitive Impairment: Results from the Wisconsin Registry for Alzheimer’s Prevention. Front. Aging Neurosci..

[35] Land W.H., Schaffer J.D. (2016). A Machine Intelligence Designed Bayesian Network Applied to Alzheimer’s Detection Using Demographics and Speech Data. Procedia Comput. Sci..

[36] König A., Satt A., Sorin A., Hoory R., Toledo-Ronen O., Derreumaux A., Manera V., Verhey F., Aalten P., Robert P.H. (2015). Automatic Speech Analysis for the Assessment of Patients with Predementia and Alzheimer’s Disease. Alzheimer’s Dement. Diagn. Assess. Dis. Monit..

[37] König A., Satt A., Sorin A., Hoory R., Derreumaux A., David R., Robert P.H. (2018). Use of Speech Analyses within a Mobile Application for the Assessment of Cognitive Impairment in Elderly People. Curr. Alzheimer Res..

[38] König A., Linz N., Tröger J., Wolters M., Alexandersson J., Robert P. (2018). Fully Automatic Speech-Based Analysis of the Semantic Verbal Fluency Task. Dement. Geriatr. Cogn. Disord..

[39] Mirzaei S., El Yacoubi M., Garcia-Salicetti S., Boudy J., Kahindo C., Cristancho-Lacroix V., Kerhervé H., Rigaud A.S. (2018). Two-Stage Feature Selection of Voice Parameters for Early Alzheimer’s Disease Prediction. Irbm.

[40] Rentoumi V., Paliouras G., Danasi E., Arfani D., Fragkopoulou K., Varlokosta S., Papadatos S. Automatic Detection of Linguistic Indicators as a Means of Early Detection of Alzheimer’s Disease and of Related Dementias: A Computational Linguistics Analysis. Proceedings of the 2017 8th IEEE International Conference on Cognitive Infocommunications (CogInfoCom).

[41] Gosztolya G., Vincze V., Tóth L., Pákáski M., Kálmán J., Hoffmann I. (2019). Identifying Mild Cognitive Impairment and Mild Alzheimer’s Disease Based on Spontaneous Speech Using ASR and Linguistic Features. Comput. Speech Lang..

[42] Beltrami D., Calzà L., Gagliardi G., Ghidoni E., Marcello N., Favretti R.R., Tamburini F. (2016). Automatic Identification of Mild Cognitive Impairment through the Analysis of Italian Spontaneous Speech Productions. Proceedings of the Proceedings of the Tenth International Conference on Language Resources and Evaluation (LREC’16).

[43] Chien Y.-W., Hong S.-Y., Cheah W.-T., Yao L.-H., Chang Y.-L., Fu L.-C. (2019). An Automatic Assessment System for Alzheimer’s Disease Based on Speech Using Feature Sequence Generator and Recurrent Neural Network. Sci. Rep..

[44] Qiao Y., Xie X.-Y., Lin G.-Z., Zou Y., Chen S.-D., Ren R.-J., Wang G. (2020). Computer-Assisted Speech Analysis in Mild Cognitive Impairment and Alzheimer’s Disease: A Pilot Study from Shanghai, China. J. Alzheimer’s Dis..

[45] Toledo C.M., Aluísio S.M., dos Santos L.B., Brucki S.M.D., Trés E.S., de Oliveira M.O., Mansur L.L. (2018). Analysis of Macrolinguistic Aspects of Narratives from Individuals with Alzheimer’s Disease, Mild Cognitive Impairment, and No Cognitive Impairment. Alzheimer’s Dement. Diagn. Assess. Dis. Monit..

[46] López-de-Ipiña K., Alonso-Hernández J.B., Solé-Casals J., Travieso-González C.M., Ezeiza A., Faúndez-Zanuy M., Calvo P.M., Beitia B. (2015). Feature Selection for Automatic Analysis of Emotional Response Based on Nonlinear Speech Modeling Suitable for Diagnosis of Alzheimer’s Disease. Neurocomputing.

[47] Lopéz-de-Ipiña K., Martinez-de-Lizarduy U., Calvo P.M., Mekyska J., Beitia B., Barroso N., Estanga A., Tainta M., Ecay-Torres M. (2017). Advances on Automatic Speech Analysis for Early Detection of Alzheimer Disease: A Non-Linear Multi-Task Approach. Curr. Alzheimer Res..

[48] Solé-Casals J., Lopéz-de-Ipiña K., Eguiraun H., Alonso J.B., Travieso C.M., Ezeiza A., Barroso N., Ecay-Torres M., Martinez-Lage P., Beitia B. (2015). Feature Selection for Spontaneous Speech Analysis to Aid in Alzheimer’s Disease Diagnosis: A Fractal Dimension Approach. Comput. Speech Lang..

[49] Martínez-Sánchez F., Meilán J.J.G., Carro J., Ivanova O. (2018). A Prototype for the Voice Analysis Diagnosis of Alzheimer’s Disease. J. Alzheimer’s Dis..

[50] Fraser K.C., Lundholm Fors K., Kokkinakis D. (2019). Multilingual Word Embeddings for the Assessment of Narrative Speech in Mild Cognitive Impairment. Comput. Speech Lang..

[51] Fraser K.C., Lundholm Fors K., Eckerström M., Öhman F., Kokkinakis D. (2019). Predicting MCI Status From Multimodal Language Data Using Cascaded Classifiers. Front. Aging Neurosci..

[52] Themistocleous C., Eckerström M., Kokkinakis D. (2018). Identification of Mild Cognitive Impairment From Speech in Swedish Using Deep Sequential Neural Networks. Front. Neurol..

[53] Khodabakhsh A., Yesil F., Guner E., Demiroglu C. (2015). Evaluation of Linguistic and Prosodic Features for Detection of Alzheimer’s Disease in Turkish Conversational Speech. Eurasip J. Audio Speech Music Processing.

[54] Khodabakhsh A., Kuscuoglu S., Demiroglu C. Detection of Alzheimer’s Disease Using Prosodic Cues in Conversational Speech. Proceedings of the 2014 22nd Signal Processing and Communications Applications Conference (SIU).

[55] Khodabakhsh A., Demiroglu C. Analysis of Speech-Based Measures for Detecting and Monitoring Alzheimer’s Disease. In Data Mining in Clinical Medicine; 2015; Volume 1246, pp. 159–173 ISBN 9781493919857.

[56] Neuberger T., Gyarmathy D., Gráczi T.E., Horváth V., Gósy M., Beke A., Sojka P., Horák A., Kopeček I., Pala K. (2014). Development of a Large Spontaneous Speech Database of Agglutinative Hungarian Language. Proceedings of the Text, Speech and Dialogue.

[57] Mar J., Arrospide A., Soto-Gordoa M., Machón M., Iruin Á., Martinez-Lage P., Gabilondo A., Moreno-Izco F., Gabilondo A., Arriola L. (2020). Validity of a Computerised Population Registry of Dementia Based on Clinical Databases. Neurología (Engl. Ed.).

[58] Johnson S.C., Koscik R.L., Jonaitis E.M., Clark L.R., Mueller K.D., Berman S.E., Bendlin B.B., Engelman C.D., Okonkwo O.C., Hogan K.J. (2018). The Wisconsin Registry for Alzheimer’s Prevention: A Review of Findings and Current Directions. Alzheimers Dement (Amst.).

[59] Hoffmann I., Nemeth D., Dye C.D., Pákáski M., Irinyi T., Kálmán J. (2010). Temporal Parameters of Spontaneous Speech in Alzheimer’s Disease. Int J. Speech Lang Pathol.

[60] Horley K., Reid A., Burnham D. (2010). Emotional Prosody Perception and Production in Dementia of the Alzheimer’s Type. J. Speech Lang. Hear Res..

[61] Hernández-Domínguez L., Ratté S., Sierra-Martínez G., Roche-Bergua A. (2018). Computer-Based Evaluation of Alzheimer’s Disease and Mild Cognitive Impairment Patients during a Picture Description Task. Alzheimers Dement (Amst).

[62] Land W.H., Schaffer J.D., Land W.H., Schaffer J.D. (2020). Alzheimer’s Disease and Speech Background. The Art and Science of Machine Intelligence: With An Innovative Application for Alzheimer’s Detection from Speech.

[63] Mueller K.D., Hermann B., Mecollari J., Turkstra L.S. (2018). Connected Speech and Language in Mild Cognitive Impairment and Alzheimer’s Disease: A Review of Picture Description Tasks. J. Clin. Exp. Neuropsychol..

[64] Kalapatapu P., Goli S., Arthum P., Malapati A. (2016). A Study on Feature Selection and Classification Techniques of Indian Music. Procedia Comput. Sci..

[65] Yahyaoui’s A., Yahyaoui I., Yumuşak N. (2018). Machine Learning Techniques for Data Classification. Advances in Renewable Energies and Power Technologies.

[66] Orimaye S.O., Wong J.S.M., Golden K.J., Wong C.P., Soyiri I.N. (2017). Predicting Probable Alzheimer’s Disease Using Linguistic Deficits and Biomarkers. BMC Bioinform..

[67] Carvajal G., Maucec M., Cullick S. (2018). Components of Artificial Intelligence and Data Analytics. Intelligent Digital Oil and Gas Fields.

[68] Capozzoli A., Cerquitelli T., Piscitelli M.S. (2016). Enhancing Energy Efficiency in Buildings through Innovative Data Analytics Technologiesa. Pervasive Computing.

[69] Hoffman J.I.E. (2019). Logistic Regression. Basic Biostatistics for Medical and Biomedical Practitioners.

[70] Stanimirova I., Daszykowski M., Walczak B. (2013). Robust Methods in Analysis of Multivariate Food Chemistry Data. Data Handling in Science and Technology.

[71] Siau K. (2003). E-Creativity and E-Innovation. The International Handbook on Innovation.

[72] Guo Z., Ling Z., Li Y. (2019). Detecting Alzheimer’s Disease from Continuous Speech Using Language Models. J. Alzheimers Dis..

